# RITA (Reactivating p53 and Inducing Tumor Apoptosis) is efficient against *TP53*^*abnormal*^ myeloma cells independently of the p53 pathway

**DOI:** 10.1186/1471-2407-14-437

**Published:** 2014-06-14

**Authors:** Sylvanie Surget, Géraldine Descamps, Carole Brosseau, Vincent Normant, Sophie Maïga, Patricia Gomez-Bougie, Nadège Gouy-Colin, Catherine Godon, Marie C Béné, Philippe Moreau, Steven Le Gouill, Martine Amiot, Catherine Pellat-Deceunynck

**Affiliations:** 1 CRCNA, INSERM, UMR 892, Nantes F-44000, France; 2 Université de Nantes, Nantes F-44000, France; 3 CNRS, UMR 6299, Nantes F-44000, France; 4 Service d'Hématologie, CHU Nantes, Nantes F-44000, France; 5 Laboratoire d'Hématologie, CHU Nantes, Nantes F-44000, France

**Keywords:** Myeloma, p53, RITA, Nutlin3a

## Abstract

**Background:**

The aim of this study was to evaluate the efficacy of the p53-reactivating drugs RITA and nutlin3a in killing myeloma cells.

**Methods:**

A large cohort of myeloma cell lines (n = 32) and primary cells (n = 21) was used for this study. This cohort contained cell lines with various *TP53* statuses and primary cells with various incidences of deletion of chromosome 17. Apoptosis was evaluated using flow cytometry with Apo2.7 staining of the cell lines or via the loss of the myeloma-specific marker CD138 in primary cells. Apoptosis was further confirmed by the appearance of a subG1 peak and the activation of caspases 3 and 9. Activation of the p53 pathway was monitored using immunoblotting via the expression of the p53 target genes p21, Noxa, Bax and DR5. The involvement of p53 was further studied in 4 different p53-silenced cell lines.

**Results:**

Both drugs induced the apoptosis of myeloma cells. The apoptosis that was induced by RITA was not related to the *TP53* status of the cell lines or the del17p status of the primary samples (p = 0.52 and p = 0.80, respectively), and RITA did not commonly increase the expression level of p53 or p53 targets (Noxa, p21, Bax or DR5) in sensitive cells. Moreover, silencing of p53 in two *TP53*^*mutated*^ cell lines failed to inhibit apoptosis that was induced by RITA, which confirmed that RITA-induced apoptosis in myeloma cells was p53 independent. In contrast, apoptosis induced by nutlin3a was directly linked to the *TP53* status of the cell lines and primary samples (p < 0.001 and p = 0.034, respectively) and nutlin3a increased the level of p53 and p53 targets in a p53-dependent manner. Finally, we showed that a nutlin3a-induced DR5 increase (≥1.2-fold increase) was a specific and sensitive marker (p < 0.001) for a weak incidence of 17p deletion within the samples (≤19%).

**Conclusion:**

These data show that RITA, in contrast to nutlin3a, effectively induced apoptosis in a subset of MM cells independently of p53. The findings and could be of interest for patients with a 17p deletion, who are resistant to current therapies.

## Background

The tumor suppressor protein p53 is a well-known major regulator of cellular stress. As a transcription factor, p53 modulates gene expression to either promote stressed cells to survive and overcome the stresses or enter cell death programs, depending on the nature and intensity of the stresses [1]. *TP53* is the most frequently mutated gene in cancers, and those mutations are associated with resistance to therapy in numerous cancers, including hematologic malignancies such as multiple myeloma (MM) [2,3]. Although MM is an incurable plasma cell malignancy, treatments have progressed in the past decade [4]. Over the last 15 years, patients at diagnosis with a deletion of the short arm of chromosome 17, del(17p), which overlaps the *TP53* locus (17p13), have been shown to have a shorter survival time that is independent of the treatment regimens [4-8]. Moreover, the frequency of del(17p) increases with successive relapses, suggesting *in vivo* selection and resistance of del(17p) ^+^ cells to therapy [9]. The incidence of the *TP53* mutation on the remaining allele is high in patients with del(17p), which suggests that *TP53* is the target gene of the chromosomal deletion [10]. Therapies that either bypass the defective p53 pathway or reactivate the p53 protein in cells expressing a mutant protein are needed. Molecules that can reactivate cell death in p53-mutant cells in a p53-dependent manner have been selected based on their ability to either kill the cells (phenotypic screening) or bind to the mutated p53 protein and restore a functional p53 conformation (biochemical screening) [11,12]. Thus, several molecules, such as PRIMA, RITA and CP-31398, have been selected and will be evaluated in clinical trials [11-15]. RITA (Reactivating p53 and inducing tumor apoptosis) was isolated from a chemical library by its ability to kill the HCT116 cell line and spare its variant, HCT116 *TP53*-/-, that lacked p53 expression [16]. Similar to nutlin3a, RITA prevents the interaction between p53 and its E3 ligase MDM2, but in contrast to nutlin3a, RITA binds to p53 and not to MDM2 [11,12]. RITA also binds to mutant p53 and reactivates some p53 functions [17]. In MM, RITA has been reported to kill myeloma cells through several pathways and to synergize with nutlin3a [18-20]. In the present work, we evaluated the efficacy of RITA in MM cells using a large collection of 32 human myeloma cell lines (HMCLs), which is representative of myeloma heterogeneity [21]. This collection is also representative of the *TP53* abnormalities found in patients (e.g., chromosome 17p deletion, different point mutations, exon deletion), which allows us to provide an accurate, preclinical evaluation. The efficacy of RITA was compared with that of nutlin3a, which reactivates the p53 pathway and only induces cell death in *TP53*^wt^ HMCLs [22]. We show that RITA, in contrast to nutlin3a, killed HMCLs and primary myeloma cells independently of the *TP53* status.

## Methods

### Human myeloma cell lines (HMCLs) and primary myeloma cells

All HMCLs used in this article were previously extensively characterized [21,23]. The HMCLs BCN, MDN, NAN-1,-3,-6,-7,-8 SBN and XG-1,-2,-3,-5,-6,-7,-11 were derived in the Nantes or Montpellier laboratories in the presence of IL-6. KMS-11, KMS12-BM, KMS12-PE and KMM1 were kindly provided by Dr Otsuki (Kawasaki Medical School, Kurashiki, Japan). ANBL-6, JJN3, JIM3, Karpas620, and MM1S HMCLs were kindly provided by Dr Jelinek (Rochester, MN, USA), by Dr. Van Riet (Brussels, Belgium), by Dr MacLennan (Birmingham, UK), by Dr Karpas (Cambridge, UK), and by Dr S. Rosen (Chicago, IL, USA), respectively. AMO1, LP1, L363, NCI-H929, SKMM2, U266 and OPM2 were purchased from DSMZ (Braunsweig, Germany), and RPMI8226 was purchased from ATTC (USA). ANBL-6, BCN, MDN, NAN, SBN and XG cells were cultured in RPMI1640 containing 5% FCS in the presence of 3 ng/ml IL6 (Novartis Pharmaceuticals, Basel, Switzerland). Blood or bone marrow samples from patients with MM at diagnosis or relapse were collected after informed consent at the Department of Hematology at the University Hospital of Nantes or at the Intergroupe Francophone du Myélome (ethical approval n° DC-2011-1399, Pr Rodat). Plasma cells were obtained after gradient density centrifugation using Ficoll-Hypaque and purification using CD138 immunomagnetic beads (Stemcell Technologies, Le Plessis Robinson, France). In all cases, the purity of the plasma cells was higher than 90%, as assessed by morphology or CD138 staining. Purified cells were cultured for 24 h in RPMI1640 containing 5% FCS and 3 ng/ml IL-6. The translocation of chromosome 14 (14q32) and deletion of chromosome 17p were assessed using FISH [10].

### Reagents and antibodies

RITA and nutlin3a were purchased from Santa Cruz Technology (Santa Cruz, CA, USA) and Sigma (Saint-Quentin Fallavier, France), respectively. Anti-Apo2.7-PE, anti-CD138-PE, control IgG1-PE and anti-BrdU-FITC mAbs were purchased from BD Biosciences (Le Pont de Claix, France). Anti-DR5-PE was purchased from eBioscience. Annexin V was purchased from Beckman Coulter (Immunotech, Marseilles, France).

### Cell death assays

Cell death in HMCLs was assessed using flow cytometry with the combined analysis of APO2.7 staining and the altered cellular morphology characteristics of apoptosis (lower FSC-H and higher SSC-H), as described previously [24]. Cell death in primary myeloma cells was measured via the loss of CD138 staining as previously described [22]. Cell death in peripheral mononuclear cells from normal donors was measured using Annexin V staining.

### Western blotting

Protein expression was evaluated using western blotting. Antibodies against the following proteins were used: p53 (Oncogene Science, Cambridge, MA, USA), NOXA (Alexis Biochemicals, Enzo Life Sciences, Villeurbanne, France), caspase-3 (Santa Cruz Biotechnology), caspase-9 (Santa Cruz Biotechnology), cleaved caspase 9 (Cell Signalling, Saint Quentin en Yvelines, France), Bax (Immunotech, Beckman Coulter, Villepinte, France), p21 (Cell Signaling), and actin (Millipore Bioscience Research Reagents, Merck Chemicals, Lyon, France).

### Cell cycle analysis

The cell cycle was assessed using BrdU/PI staining [25]. Briefly, cells were incubated for 30 minutes with BrdU (1 mg/ml) at 37°C in culture medium, washed and fixed in 50% ethanol in PBS and froze at -20°C for 24 hours. Fixed cells were then washed, incubated for 30 minutes at 37°C in 2 M HCL, washed in 0.5% Tween20 PBS and further incubated overnight at 4°C with anti-BrdU-FITC mAb. The cells were then washed in PBS, and PI (2.5 μg/ml) was added prior to immediate fluorescence analysis using a flow cytometer (FACSCalibur, Becton Dickinson).

### TP53 silencing

Stably modified myeloma cell lines were obtained via lentiviral cell transduction as previously described [22]. Briefly, MDN, NCI-H929, XG6, XG5 and KMS12PE HMCLs were transduced with a lentivirus carrying shRNA control or an shRNA that was designed to knock down p53 (CCGGGTCCAGATGAAGCTCCCAGAACTCGAGTTCTGGGAGCTTCATCTGGACTTTTT, RefSeq NM_000546; Sigma Aldrich). Cells were plated in 24-well plate (200,000 cells/ml) and infected with lentivirus (MOI = 2). Selection with puromycin (4 μg/mL) was begun 4 days after infection, and all silenced HMCLs were stable and cultured under the continuous selection of puromycin.

### Statistical analysis

Statistical analyses were performed using the Kruskal-Wallis, Anova, Mann–Whitney, Fisher exact and Wilcoxon matched-pairs signed rank tests, as indicated within the text.

## Results

### HMCLs displayed wide, heterogeneous sensitivity to RITA

**Figure 1:**
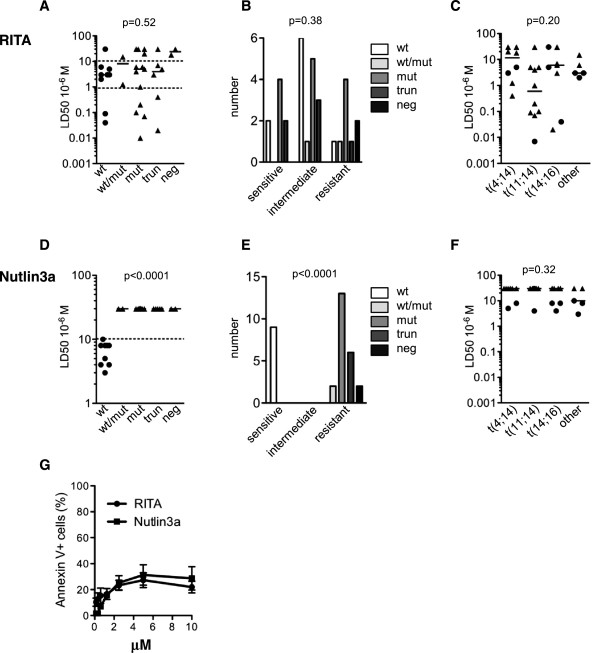
**Myeloma cells were heterogeneously sensitive to RITA. A,D**. LD_50_ values for RITA **(A)** and nutlin3a **(D)** of HMCLs were plotted against the *TP53* status of HMCLs. wt: wild type, m: mutated, trunc: truncated, KO: deleted**.** Cells (100,000/0.2 ml) from 32 HMCLs were incubated for 72 h with serial dilutions of RITA (20 μM to 1 nM) or nutlin3a (20 μM to 1.25 μM), and cell death was assessed using flow cytometry after APO2.7 staining (Cell Quest software, FACSCalibur). The LD_50_ was defined as the concentration that killed 50% of cells (mean of 3 experiments). wt: wild-type, m:mutated, trun: truncated, KO: deleted.** B,E**. Contingency analysis of the sensitivity of HMCLs to RITA **(B)** or nutlin3a **(E)** with respect to *TP53* status. **C,F**. The LD_50_ values for RITA **(C)** and nutlin3a **(F)** of HMCLs were plotted against the 14q32 status of the HMCLs. Circle and triangle symbols represent *TP53*^*wt*^ and *TP53*^*Abnormal*^ HMCLs, respectively. **G**. Apoptosis induced by RITA and nutlin3a in normal PBMCs. PBMCs were incubated for 3 days in RPMI1640 medium containing 5% FCS with serial concentrations of RITA or nutlin3a. Data represent the mean ± SE of 3 independent donors.

RITA was first reported to kill TP53^+/+^ HCT116 cells with a low LD_50_ (50 nM) but not TP53^-/-^ HCT116 cells (LD_50_ = 50 μM) [16]. In some *TP53-*mutated cancer cells, RITA was effective, with an LD50 equal to or less than 1 μM [26]. We therefore assessed the sensitivity of myeloma cells to RITA using 32 HMCLs and serial concentrations of RITA that ranged from 1 nM to 20 μM (Table 1). Sensitivity was analyzed according to the *TP53* status of the HMCLs i.e., *TP53*^*wt*^ (n = 9), *TP53*^*wt/mutated*^ (n = 2), *TP53*^*mutated*^ (n = 13), *TP53*^*truncated*^ (n = 6) or *TP53*^*negative*^ (n = 2) as previously described [21,22]. The LD_50_ values were heterogeneous and ranged from 7 nM to more than 20 μM (Figure 1A). For 6 HMCLs, the LD_50_ was not obtained at 20 μM and was arbitrarily considered to be 30 μM to perform statistical analyses. The LD_50_ values of HMCLs in the 5 *TP53* groups were not significantly different (Figure 1A, p = 0.52, Kruskal-Wallis test). The HMCLs were considered resistant when the LD_50_ was greater than 10 μM, intermediate when the LD_50_ was between 1 and 10 μM and sensitive when the LD_50_ was less than 1 μM. Thus, 9 HMCLs were resistant, 15 were intermediate and 8 were sensitive (2 *TP53*^*wt*^, 4 *TP53*^*mutated*^ and 2 *TP53*^*truncated*^), with no significant difference them according to the *TP53* status (p = 0.38, Chi-2 test, Table 1 and Figure 1B). The heterogeneous sensitivity of the HMCLs to RITA (Figure 1C) was not related to the molecular heterogeneity of the 14q32 translocation (p = 0.2, Anova test).

**Table 1:** HMCLs’ characteristics and sensitivity to RITA and nutlin3a

	**HMCLs’ characteristics**			**RITA μM**		**Nutlin3a μM**	
	**HMCL**	***TP53 *** **cDNA**	**t()**	**LD**_**50**_	**SD**	**LD**_**50**_	**SD**
TP53^wild-type^	MM1S	wt	t(14;16)	0.04	0.01	8	2
	MDN	wt	t(11;14)	0.09	0.05	4	1
	SBN	wt	t(14 ;?)	2	0.5	3	1
	NCI-H929	wt	t(4;14)	3	0.5	8	2
	AMO1	wt	t(12;14)	3	0.5	8	2
	XG3	wt	none	3	0.5	10	2
	XG7	wt	t(4;14)	5	1	5	1
	XG6	wt	t(14;16)	5	1	4	1
	BCN	wt	t(14;16)	> > 20		8	2
TP53^negative^	KMS11	No expression	t(4;14)	18	3	> > 10	
	JJN3	No expression	t(14;16)	> > 20		> > 10	
TP53^wt+mutated^	KMM1	C135C + F	t(6;14)	15	3	> > 10	
	NAN3	R248R + Q	t(4;14)	1.5	0.5	> > 10	
TP53^mutated^	XG5	R282W	t(11;14)	0.007	0.004	> > 10	
	KMS12BM	R337L	t(11;14)	0.07	0.03	> > 10	
	KMS12PE	R337L	t(11;14)	0.1	0.05	> > 10	
	SKMM2	K132N	t(11;14)	0.2	0.1	> > 10	
	XG1	Y126N	t(11;14)	1	0.2	> > 10	
	XG11	C135Y	t(11;14)	4	1	> > 10	
	KARSPAS620	C135Y	t(11;14)	5	1	> > 10	
	RPMI8226	E285K	t(14;16)	6	1	> > 10	
	XG2	C176Y	t(12;14)	6	1	> > 10	
	JIM3	R273C	t(4;14)	20	3	> > 10	
	OPM2	R175H	t(4;14)	> > 20		> > 10	
	LP1	E286K	t(4;14)	> > 20		> > 10	
	U266	A161T	t(11;14)	> > 20		> > 10	
TP53^truncated^	NAN6	exons7-9^€^	t(14;20)	0.02	0.01	> > 10	
	NAN8	exon7*	t(4;14)	0.4	0.1	> > 10	
	NAN1	E180STOP	t(14;16)	3	1	> > 10	
	NAN7	exon7*	t(11;14)	5	2	> > 10	
	L363	intron 7^$^	t(20;22)	7	2	> > 10	
	ANBL6	Q331STOP	t(14;16)	> > 20		> > 10	

HMCLs’ characteristics were previously reported (21). RITA and nutlin3a LD_50_ values (dose that killed 50% of cells in 72 h) were determined as described within the Methods section. SD: standard deviation. *TP53* cDNA sequencing was performed after 2 overlapping PCR assays. HMCLs displaying *TP53* mRNA sequence lacking exons (NAN6, NAN7, NAN8) or displaying mutation impairing exon splicing (L363) or nonsense mutations (NAN1 and ANBL-6) were gathered and called “truncated”. ^€^lack of exons 7, 8 and 9. *no amplification after exon 7. ^$^L363 cells displayed a point mutation in exon7/intron 7 splicing junction (base C782G) impairing splicing of intron 7.

In contrast, all *TP53*^*wt*^ HMCLs were killed by the MDM2 inhibitor nutlin3a, with LD_50_ values ranging from 3 to 10 μM, while none of the LD_50_ values for any *TP53*^*Abnormal*^ of the HMCLs were reached at 20 μM, so were arbitrarily considered to be 30 μM (p < 0.0001, Kruskal-Wallis test, Figure 1D) [22]. Thus, all *TP53*^*wt*^ HMCLs were sensitive and all *TP53*^*Abnormal*^ HMCLs were resistant to nutlin3a (Figure 1E). These data showed that RITA, in contrast to nutlin3a, killed myeloma cells independently of their *TP53* status. The heterogeneous sensitivity of the HMCLs to nutlin3a (Figure 1F) was not related to the molecular heterogeneity (p = 0.32, Anova test).To assess the toxicity of each drug against normal cells, peripheral blood mononuclear cells (PBMCs) from 3 independent donors were incubated for 3 days with serial concentrations of each drug. RITA (1.25 μM) did not induce any significant apoptosis in PBMCs (16% ± 4% Annexin V + cells), while nutlin3a (10 μM) did (29% ± 9%), Figure 1G.

#### RITA did not increase the expression of p53 targets such as p21 and Bax or induce p53 translocation to mitochondria

**Figure 2:**
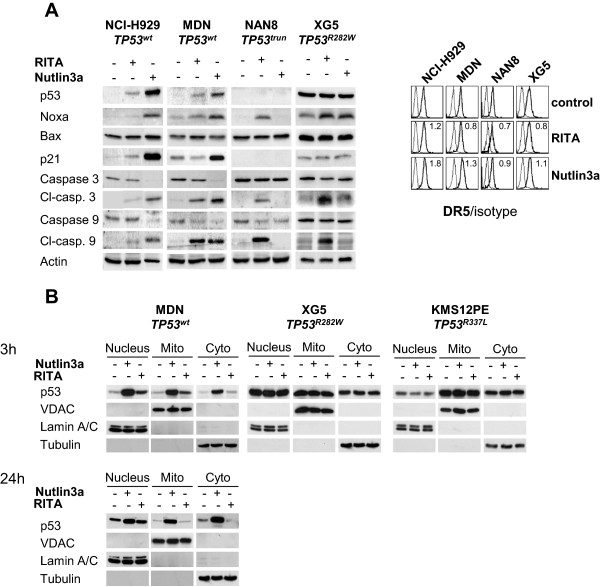
**RITA did not increase the expression of p53 targets in HMCLs. A**. Cells were incubated for 24 hours with different concentrations of RITA or nutlin3a i.e., 150 nM and 4 μM in MDN, respectively, 3 μM and 10 μM in NCI-H929, respectively, 600 nM and 10 μM in NAN8, respectively, and, 20 nM and 10 μM in XG5. Cells were harvested and either lysed following protein expression analysis using western blotting as indicated in the figure, or stained with anti-DR5-PE or control-PE mAbs, and the fluorescence was analyzed using a FACSCalibur. One representative experiment of 3 is shown. DR5 expression fold-change (i.e., DR5 ratio of RITA- or nutlin3a-treated cells divided by ratio of control cells) induced by RITA or nutlin3a is indicated within cytograms. **B**. Cells were incubated for 3 h (or 24 h) with 10 nM (XG5) or 100 nM (MDN, KMS12PE) RITA or nutlin3a (5 μM for MDN and 1 μM for KMS12PE and XG5). The proteins from the nucleus, mitochondria and cytosol were extracted using the Cell Fractionation Kit-Standard (Abcam, Cambridge, UK).

Because RITA reactivates wild-type and mutated p53 proteins, we assessed whether RITA increased the expression of p53 and p53 targets involved in the intrinsic or extrinsic pathways of apoptosis, i.e., Bax, Noxa, p21 and DR5, using Western blotting and flow cytometry. RITA slightly increased the expression of p53 in *TP53*^*wt*^ NCI-H929 and MDN. Except for DR5, expression of which was slightly increased in NCI-H929 only (1.26-fold increase), RITA failed to significantly increase the expression of p21 or Bax in sensitive *TP53*^*wt*^ or *TP53*^*Abn*^ cell lines but increased that of Noxa in all HMCLs (Figure 2A). These results showed that an increase in expression of the p53 targets p21, Bax and DR5, was not a hallmark of cell response to RITA. In contrast, nutlin3a strongly increased the expression of p53, Noxa and p21 in *TP53*^*wt*^ NCI-H929 and MDN cells, but not in *TP53*^*Abn*^ NAN8 and XG5 cells. Cell death that was induced by RITA or nutlin3a correlated with the cleavage of caspases 3 and 9.

In response to several stresses, such as p53 stabilization or oxidative stress, p53 translocates to the mitochondria, and this early translocation triggers apoptosis independently of the p53-mediated transcriptional increase in pro-apoptotic gene expression [27,28]. We therefore analyzed the p53 level and localization in *TP53*^*wt*^ MDN, *TP53*^*mutated*^ KMS12PE and XG5 HMCLs, which were all sensitive to RITA. As shown in Figure 2B, nutlin3a induced a rapid (3 h) and long-lasting increase in p53 levels (the increase was still detectable after 24 h, lower panel) in all fractions of *TP53*^*wt*^ MDN cells (Figure 2B). In contrast, RITA slightly increased the p53 levels in the mitochondrial and nuclear fractions after 3 h (Figure 2B, upper panel), and this increase was no longer detectable after 24 h (Figure 2B, lower panel). In *TP53*^*mutated*^ XG5 cells and KMS12PE, nutlin3a and RITA had no effect on the p53 level or localization (Figure 2B). Although these data did not exclude any roles of p53 in RITA-induced cell death in MDN cells, they did not favor p53 involvement in RITA-induced cell death in XG5 or KMS12PE cells.

### p53-silenced HMCLs remained highly sensitive to RITA

**Figure 3:**
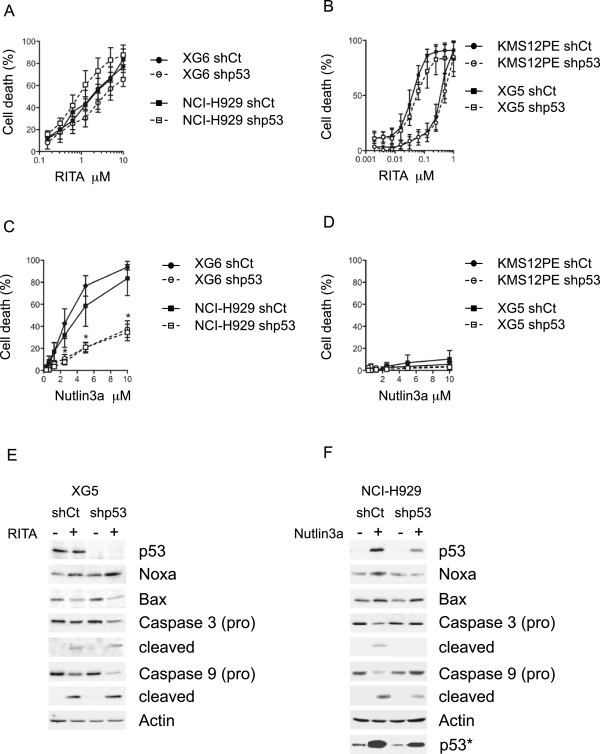
**Silencing p53 did not inhibit the myeloma cell death induced by RITA. A,D** : Dose responses of shCt and shp53 TP53^wt^ or TP53^mut^ cells to RITA **(A,B)** and nutlin3a **(C,D)** were evaluated as described in the legend of Figure 1. For RITA, the LD_50_ values (μM) of shCt and shp53 cells were 0.20 ± 0.1 and 0.23 ± 0.03 for KMS12PE, 0.04 ± 0.01 and 0.06 ± 0.01 for XG5, 1.9 ± 0.8 and 2.7 ± 2 for XG6 and 1.8 ± 0.5 and of 1.5 ± 0.5 for NCI-H929, respectively. For nutlin3a, the LD_50_ values (μM) of shCt and shp53 cells were 3.9 ± 2.4 and >10 for NCI-H929, 3.1 ± 1.4 and >10 for XG6 and >10 for KMS12PE and XG5, respectively. *indicates a significant difference between shCt and shp53 HMCls (p < 0.05). **E, F**: Western blot analysis of p53, Noxa, Bax and caspase expression in shCt and shp53 XG5 cells treated overnight with 100 nM RITA **(E)** and in shCt and shp53 NCI-H929 cells treated overnight with 10 μM nutlin3a **(F)**. *indicates that membrane was overexposed to assess constitutive p53 level in shp53 and shCont cells. P53 expression was decreased by 63% and 68% in untreated and treated NCI-H929 cells, respectively. Extinction level could not be calculated in XG5 because no p53 expression could be detected in shp53 XG5 cells.

To investigate a direct role of p53 in cell death, we silenced p53 in *TP53*^*wt*^ (MDN, NCI-H929 and XG6) and *TP53*^*mutated*^ (KMS12PE and XG5) HMCLs. Shp53 MDN cells could not be obtained (data not shown), but silencing of p53 did not significantly modify the apoptosis induced by RITA in NCI-H929 and XG6 *TP53*^*wt*^ (p > 0.5, Figure 3A) or in KMS12PE and XG5 *TP53*^*mutated*^ HMCLs (p > 0.5, Figure 3B). In contrast and as expected, p53 silencing significantly increased nutlin3a LD_50_ values (p < 0.05) in NCI-H929 and XG6 *TP53*^*wt*^ HMCLs (Figure 3C) but did not modulate resistance in KMS12PE and XG5 *TP53*^*mutated*^ HMCLs (Figure 3D). The activation of caspases and decrease in Bax expression induced by RITA treatment remained unchanged in shp53 XG5 cells when compared with shCt cells while the increase in Noxa expression was maintained (Figure 3E). In contrast, the activation of caspases 3 and 9, and the increase in expression of p53 and Noxa induced by nutlin3a were inhibited in shp53 NCI-H929 cells when compared with shCont cells (Figure 3F). These data demonstrated that silencing p53 impaired the cell death that was induced by nutlin3a, but not that induced by RITA.

### RITA and nutlin3a induced apoptosis but modulated the cell cycle differently

The cell cycle of HMCLs treated with RITA or nutlin3a was analyzed using double PI/BrdU staining, as previously reported [25]. The cell cycle was analyzed in *TP53*^*wt*^ MDN cells, which were killed by both drugs, and *TP53*^*mutated*^ KMS12PE cells, which were sensitive to only RITA (Additional file 1: Figure S1). In MDN cells, RITA and nutlin3a induced a subG1 peak and an increase in cells in the G2 phase. However, nutlin3a increased but RITA decreased the proportion of cells in G1 phase. Moreover, RITA slightly decreased the proportion of cells in S phase, while nutlin3a highly inhibited it. This differential regulation of the G1 and S phases could be related to p21, which was induced by nutlin3a but not by RITA (Figure 2A). Finally, RITA and nutlin3a induced the accumulation of cells that had an intermediate DNA content between G1 and G2, but were BrdU-negative, which could be related to either a blockade of cells in S phase or the apoptosis of cells in G2 phase (subG2 peak). In KMS12PE cells (and in XG5 cells, data not shown), RITA induced a subG1 peak, decreased the proportion of cells in G1 phase, but not in S phase, and increased the proportion of cells in G2 phase, but nutlin3a had no significant effect on the cell cycle, which coincided with its inability to induce cell death. These data showed that RITA and nutlin3a differentially modulated the cell cycle and that RITA induced a similar modulation of the cell cycle in *TP53*^*wt*^ and *TP53*^*mutated*^ HMCLs.

### RITA killed primary cells but did not increase DR5 expression

**Figure 4:**
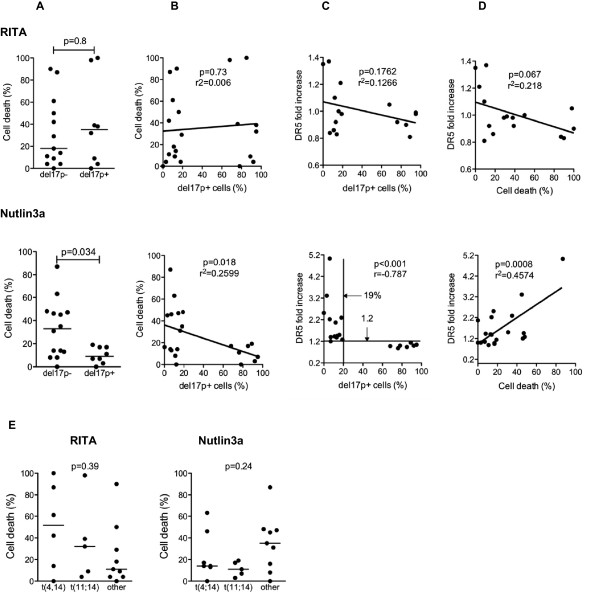
**The RITA sensitivity of primary cells was not significantly related to *TP53 *status. A.** The percentage of cell death of primary cells that were treated with RITA (upper panel) or nutlin3a (lower panel) was analyzed as a function of del(17p). del(17p)+: >19% of deleted cells; del(17p)-: ≤19% of deleted cells.** B**. The percentage of cell death of primary cells treated with RITA (upper panel) or nutlin3a (lower panel) was plotted against the percentage of del(17p) + cells within the samples.** C**. The fold increase in DR5 expression induced by RITA (upper panel) or nutlin3a (lower panel) was plotted against the percentage of del(17p) + cells within the samples.** D**. The fold increase in DR5 expression that was induced by RITA (upper panel) or nutlin3a (lower panel) was plotted against the percentage of cell death.** E**. The percentage of cell death of primary cells treated with RITA (left) or nutlin3a (right) was analyzed as a function of 14q32 translocation. Circle and triangle symbols represent samples without and with del(17p), respectively.

Purified primary myeloma cells from patients were incubated for 24 h with or without RITA (1 μM) or nutlin3a (10 μM). Cell death in 22 consecutive samples was then evaluated based on the loss of CD138 expression, and DR5 staining was performed to monitor the p53 response, as previously described [22]. Samples were assessed for the deletion of chromosome 17p using FISH. In samples, the incidence of del(17p) involved a minor (<20%) or a major (> > 50%) fraction of cells, Table 2. Thus, samples with up to 19% deleted cells (n = 14) were considered to be negative for the deletion, while those with at least 68% deleted cells were considered to be positive (n = 8). Primary cells were heterogeneously sensitive to RITA with a proportion of induced cell death that ranged from 0% to 95% (median value 23.5%, n = 21), but no correlation with del(17p) was observed. The proportion of cell death that was induced by RITA was not significantly different in samples with or without del(17p) (median cell death 35% versus 18%, respectively, p = 0.80, Mann–Whitney test, Figure 4A), and no direct correlation was found between the percentages of cell death and del(17p) (p = 0.73, Figure 4B). RITA failed to increase DR5 expression in samples (median fold increase of 0.98, n = 15, p = 0.9, Wilcoxon matched-pairs signed rank test), and no linear correlation between DR5 modulation and the proportion of cells with del(17p) was observed (p = 0.18, Pearson test, Figure 4C). However, RITA had a trend to decrease DR5 expression in sensitive cells (p = 0.07, Pearson test, Figure 4D). As observed in HMCLs, the sensitivity of primary cells to RITA was not related to the t(4;14) or t(11;14) translocations (p = 0.39, Anova test, Figure 4E).

**Table 2:** Sensitivity of primary cells to RITA and nutlin3a

**Samples’ characteristics**							**Cell death**		**DR5 fold increase**	
**Number**	**Disease**	**Status**	**Origin**	**t(4;14)**	**t(11;14)**	**del(17p)**	**RITA**	**Nutlin3a**	**RITA**	**Nutlin3a**
1	pPCL	D	PB	-	-	0%	0%	16%	1.35	2.51
2	MM	R	BM	-	-	3%	4%	45%	nd	3.31
3	MM	D	BM	+	-	6%	42%	14%	nd	2.22
4	sPCL	R	PB	-	-	6%	11%	87%	1.37	5.03
5	MM	D	BM	+	-	7%	nd	46%	nd	1.20
6	MM	D	BM	+	-	7%	87%	13%	0.84	1.40
7	MM	R	BM	+	-	10%	61%	63%	nd	nd
8	MM	D	BM	-	-	11%	18%	8%	0.86	1.43
9	MM	D	BM	-	-	12%	9%	0%	1.10	2.07
10	MM	D	BM	+	-	13%	14%	14%	0.92	1.38
11	MM	D	BM	-	-	14%	90%	47%	0.83	1.46
12	MM	D	BM	-	-	16%	50%	31%	1.00	1.50
13	MM	R	BM	-	-	18%	4%	35%	1.21	2.29
14	sPCL	R	PB	-	-	19%	29%	48%	0.98	1.28
15	sPCL	R	PB	-	+	68%	98%	17%	1.05	0.98
16	MM	D	BM	-	+	76%	39%	11%	0.92	0.88
17	MM	D	BM	+	-	78%	0%	0%	nd	1.00
18	pPCL	D	PB	+	-	85%	100%	17%	0.90	0.94
19	pPCL	D	PB	-	+	89%	9%	19%	0.81	1.14
20	MM	R	BM	-	+	92%	4%	3%	nd	1.00
21	pPCL	R	PB	-	+	95%	32%	7%	0.99	1.05
22	sPCL	R	PB	-	nd	95%	38%	7%	0.98	1.04

The cells were treated overnight with RITA (1 μM) or nutlin3a (10 μM). The cell death and DR5 expression were assessed by flow cytometry, respectively by the loss of CD138 expression or direct DR5 staining. The DR5 fold increase was calculated by dividing the mean fluorescence ratios (specific over control staining) of treated cells by that of untreated cells. The chromosome 17p deletion and t(14q32) translocations were assessed by FISH.

MM: multiple myeloma, pPCL: primary plasma cell leukemia, sPCL: secondary plasma cell leukemia, D: diagnosis, R: relapse, BM: bone marrow, PB: peripheral blood, nd: not done.

In contrast, nutlin3a significantly induced cell death in samples without del(17p), but not in samples with del(17p): the median cell death was 33% versus 9%, respectively (p = 0.034, Mann–Whitney test, Figure 4A). Moreover, we found a linear and negative correlation between the proportion of cell death that was induced by nutlin3a and the incidence of cells with del(17p) (p = 0.018, Pearson test, Figure 4B). Nutlin3a significantly increased DR5 expression in samples without del(17p) (median fold increase of 1.50, p = 0.0002, n = 13, Wilcoxon matched-pairs signed rank test), but not in the samples with del(17p) (median fold increase of 1, p = 0.6, n = 8, Wilcoxon matched-pairs signed rank test), and we found a negative correlation between the proportion of cells with del(17p) and an increase in DR5 (p < 0.001, Spearman test, Figure 4C). Moreover, we defined two significant cut-off values for del(17p) and DR5 modulation (see ROC curves in Additional file 2: Figure S2): all samples with up to 19% deleted cells (range 0%-19%) showed up-regulated DR5 by more than 1.2-fold (n = 13), while all samples with more than 19% deleted cells (range 68%-95%, n = 8) showed up-regulated DR5 by less than 1.2-fold, (p < 0.0001, Fisher’s exact test, sensitivity = 100%, specificity = 100%, Figure 4C). Finally, a linear correlation between the percentage of cell death and the fold-increase in DR5 expression was observed (n = 21, p = 0.0008, Figure 4D). The sensitivity of primary cells to nutlin3a was not related to the t(4;14) or t(11;14) translocations (p = 0.24, Anova test, Figure 4E).

These data obtained using primary samples showed that, in contrast to nutlin3a, RITA induced cell death independently of *TP53* status and did not up-regulate DR5 expression.

## Discussion

In this study, we compared the efficacy of two p53-reactivating drugs, nutlin3a and RITA, for inducing cell death in myeloma cells using a collection of 32 HMCLs. In this cell line collection, 9 were *TP53*^*wt*^, 15 were *TP53*^*mutated*^ (13 different mutations with or without loss of heterozygosity), 6 were *TP53*^*truncated*^ and 2 were *TP53*^*negative*^. This collection represented both myeloma specificity (chromosomal abnormalities involving the 14q32 locus) and *TP53* diversity. These different TP53 statuses allowed us not to test constitutive or non-constitutive p53 expression without overexpressing abnormal *TP53* in the *TP53*^*neg*^ cell lines, as is observed in the cells of patients. Using this collection, we showed that nutlin3a efficacy was restricted to *TP53*^*wt*^ HMCLs in a p53-dependent manner. In contrast, RITA effectively killed 25% of HMCLs independently of the *TP53* status: 22% of *TP53*^*wt*^ and 26% of *TP53*^*mutated*^ HMCLs. Silencing p53 in two *TP53*^*mutated*^ RITA-sensitive HMCLs failed to decrease RITA killing, which argued against an involvement of p53 in the cell-death response. Moreover, TP53^trunc^ NAN8 cells that did not display any p53 expression by western blot analysis (using mAb directed against the N-terminal portion of the protein, data not shown) were extremely sensitive to RITA. Cell death induced by nutlin3a or RITA involved apoptosis, as shown by the activation of caspases 3 and 9, Apo2.7 staining and a subG1 peak. In contrast to nutlin3a, RITA did not increase or induce the expression of DR5, Noxa, Bax or p21 in *TP53*^*wt*^ HMCLs. All of these data clearly indicated that RITA effectively killed 25% of HMCLs in a p53-independent manner. Of interest, RITA efficiently killed cell lines that were resistant to the alkylating drugs melphalan and bendamustine (such as KMS12PE, XG5, and NAN8) [29].

RITA and nutlin3a efficacy was also assessed in 22 primary samples (blood or bone marrow) from patients at diagnosis or relapse. As observed in the cell lines, RITA killed some primary samples independently of the presence or absence of del(17p). Additionally, RITA was not effective in increasing DR5 expression when compared with nutlin3a. However, a weak increase in DR5 level occurred in 2 samples without del(17p) (n°1 and 4) in which RITA did not induce cell death (Table 2). This weak increase suggested that RITA weakly activated the p53 pathway in these cells but was unable to induce cell death. Of note, RITA failed to increase, but rather decreased, DR5 in sensitive samples it, which argued against the involvement of the p53 pathway in cell death. The proportion of samples that were highly sensitive to RITA (50% cell death with 1 μM) was similar in the cell lines (25%) and in primary myeloma cells (29%). Notably, RITA was effective against 2 primary plasma cell leukemia samples that harbored del(17p) (n° 15 and 18), and represented an extremely aggressive presentation of MM. In CLL, Nahi et al. reported that del(17p) had no impact in cell death induced by RITA in primary cells, while AML samples with del(17p) were less sensitive to RITA when compared with samples without del(17p) [30]. Thus, at least in MM and CLL, it appears that RITA-induced cell death was not impacted by del(17p). In contrast, nutlin3a only killed myeloma primary cells lacking del(17p), and the cell death was negatively correlated to the incidence of cells with del(17p) within the sample (Figure 4). Moreover, we demonstrated a strong negative correlation between the DR5 increase and the proportion of cells with del(17p) (cut-off values of ≤19% for cell with deletions and ≥1.2 for increased-DR5). This correlation was consistent with our previous findings that showed that nutlin3a induced *DR5* mRNA expression in myeloma through the increased binding of p53 to the *DR5* gene [22]. Thus, in myeloma cells, a DR5 increase can be considered a good marker of p53 activation by nutlin3a. Reciprocally, a DR5 increase in the presence of nutlin3a can signify the re-activation of a functional p53 pathway. However, cell death was not induced in any of the samples lacking del(17p), despite the DR5 increase. This lack of detectable apoptosis could be related to the fact that the primary samples were not maintained in culture for more than 24 hours in culture (usually 15 hours) to avoid spontaneous apoptosis. This exposure was too short for an optimal cell death detection, especially death induced by nutlin3a. Indeed, cell death induced by RITA was detectable in cell lines after a 15-hour exposure, in contrast to that induced by nutlin3a, which is detectable after 24 hours. In the cell lines, the increase in DR5 expression preceded the appearance of cell death (4 to 6 hours versus 1 day). Thus, the lack of nutlin3a-induced cell death in several samples lacking del(17p), despite an increase in DR5 expression, could be due to the short incubation.

While nutlin3a widely induced apoptosis in *TP53*^*wt*^ myeloma or lymphoma cells, it was unable to kill solid cancer cell lines (HCT116, U2OS, MCF-7) [31]. Indeed, despite the nutlin3a-induced accumulation of p53, apoptotic genes were not turned on when *CDKN1A* was strongly induced. In myeloma cells, the expression of *CDKN1A* and apoptotic genes is induced, which leads to cell cycle arrest and apoptosis. This major difference could be related to either different oncogenic abnormalities (notably those targeting some actors of the MDM2/p53 regulation, such as ARF) or to the expression of p53 isoforms, which are tissue specific and impact the transcription of p53-regulated genes [32]. Thus, at least in myeloma and hematological malignancies, nutlin3a or related compounds, could have a therapeutic application.

RITA kills some *TP53*^*wt*^ HMCLs by mobilizing the p53 or JNK pathways [18,20]. Because we could not efficiently silence p53 in highly sensitive *TP53*^*wt*^ cell lines, such as MDN or MM1S, we could not exclude the possibility that RITA mobilized the p53 pathway, although none of the p53 target genes were induced. However, we can conclude that RITA kills *TP53*^*mutated*^ myeloma cells within the nanomolar range, regardless of p53 expression. Moreover, RITA also killed NAN8 cells that expressed a truncated form of *TP53* mRNA and in which the p53 protein could not be detected by western blotting. The molecular mechanism of the action of RITA remains unclear, and its ability to bind to p53 is somewhat controversial [16,33]. RITA was initially isolated from a chemical library through a phenotypic screening, i.e., by its ability to kill TP53^+/+^ HCT116 cells but not TP53^-/-^ HCT116 [16]. The p53 dependency of RITA has been widely studied in the HCT116 *TP53*^*+/+*^ and *TP53*^*-/-*^ paired cell lines, and the results have been generalized to other types of cancer cells, although the involvement of p53 involvement in all cell lines has not been demonstrated. Of note, nutlin3a does not kill HCT116 cells but induces cell-cycle arrest, in contrast to RITA, which kills the cells. A recent publication demonstrated that RITA killed Ewing sarcoma cells independently of p53 through the degradation of IGF1R [34]. This mechanism is unlikely to occur in myeloma cells because we did not observe any modulation of IGF1R expression (data not shown) and we previously showed that blocking IGF1R signaling induced cell growth arrest but not cell death [35]. RITA also induces cell death by decreasing the transcription of several oncogenes, such as *BCL2, MYC, MCL1, BIRC5* and *IGF1R*, independently of its ability to induce the expression of pro-apoptotic genes [36]. More recently, CHK2 has been shown to mediate the RITA-induced cell death in the HCT116 model, and it was shown that replicating cells were more prone to entering RITA-induced apoptosis [37]. Taking all of these mechanisms together, it appears that RITA induces or represses more than one pathway in cancer cells and that the activities of RITA are not restricted to p53. Concerning MM, the elucidation of cell death mechanism(s) in the nanomolar range in *TP53*^*mutated*^ or *TP53*^*truncated*^ and in silenced *TP53*^*mutated*^ cells requires further studies.

Both RITA and nutlin3a displayed some toxicity against normal peripheral mononuclear cells when used at high concentrations. However, the lack of data in humans precludes any definitive conclusion concerning any *in vivo* toxicity.

## Conclusion

In summary, these data show that RITA, in contrast to nutlin3a, effectively induced apoptosis in a subset of MM cells independently of p53. Although the sensitivity of cells to RITA can only be predicted using *ex vivo* testing, RITA could be of interest for patients harboring *TP53* abnormalities, who are resistant to current therapies.

## Competing interests

The author(s) declare that they have no competing interests.

## Authors’ contribution

SS participated in the design of the study and performed the experiments. GD, VN, CB, PG-B, SM and NG-C performed the experiments. CG reviewed FISH experiments. PM and SLG participated in the design of the study and collected patient samples. MCB reviewed the paper. MA participated in the design of the study and in the writing of the paper. CPD designed the study and wrote the paper. All authors read and approved the final manuscript.

## Pre-publication history

The pre-publication history for this paper can be accessed here:

http://www.biomedcentral.com/1471-2407/14/437/prepub

## Supplementary Material

Additional file 1: Figure S1 The cell cycle was analyzed by BrdU and PI staining 24 h after RITA (100nM) or nutlin3a (5 μM for MDN, 10 μM for KMS12PE) addition (see materials and methods). One representative experiment out of 2 is shown. Click here for file

Additional file 2: Figure S2 The true positive rates (sensitivity, Se) of the DR5 modulation (left) and the percentage of 17p deletion (right) were plotted in function of the false positive rate (1-specificity, 1-Sp). As indicated by the arrows, the respective 1.2 and 19% thresholds for the DR5 increase and 17p deletion provided 100% sensitivity and specificity. Click here for file
