# Supplementary material for: RITA (Reactivating p53 and Inducing Tumor Apoptosis) is efficient against TP53abnormal myeloma cells independently of the p53 pathway
Source: BMC Cancer. 2014 Jun 14;14:437. doi: 10.1186/1471-2407-14-437 (PMC4094448; doi:10.1186/1471-2407-14-437)
Supplement: Additional file 1: Figure S1 — The cell cycle was analyzed by BrdU and PI staining 24 h after RITA (100nM) or nutlin3a (5 μM for MDN, 10 μM for KMS12PE) addition (see materials and methods). One representative experiment out of 2 is shown. [file 1471-2407-14-437-S1.pdf]

Figure S1

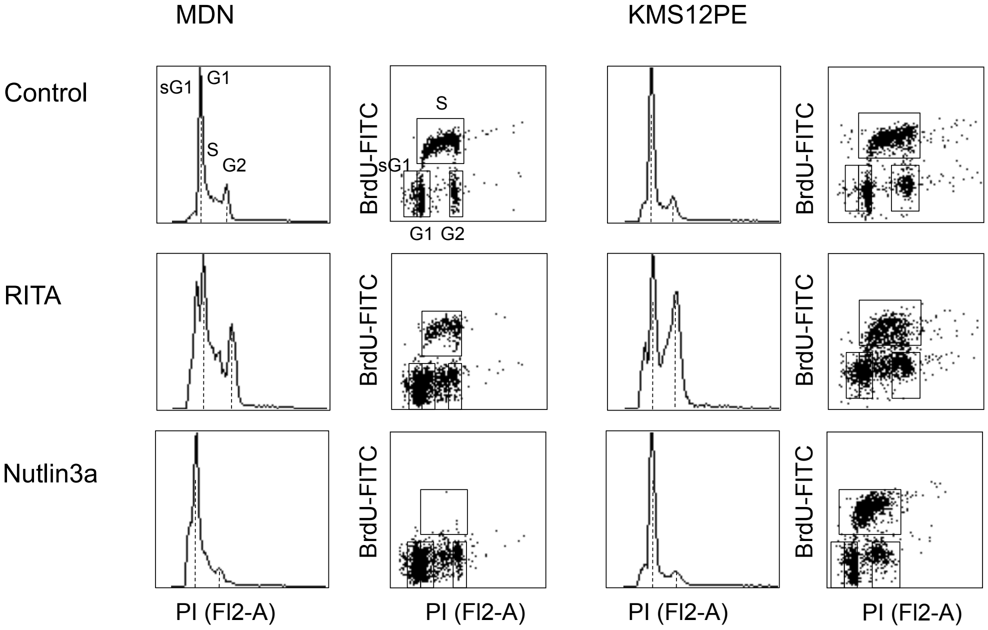


| | Control | RITA | Nutlin3a | Control | RITA | Nutlin3a |
|---|---|---|---|---|---|---|
| subG1 | 1.6 | 35.4 | 29.4 | 1.7 | 12.6 | 2.6 |
| G1 | 49.4 | 19.7 | 42.7 | 42.9 | 23.0 | 45.6 |
| S | 35.6 | 18.2 | 0.1 | 40.2 | 35.9 | 36.2 |
| G2 | 9.0 | 8.7 | 14.3 | 13.2 | 21.2 | 18.7 |
| G1-G2 | 1.4 | 18.0 | 13.4 | 2.0 | 7.2 | 0 |

Figure S1.The cell cycle was analyzed by BrdU and PI staining 24 h after RITA (100 nM) or nutlin3a (5 μM for MDN, 10 μM for KMS12PE) addition (see materials and methods). One representative experiment out of 2 is shown.
