# Supplementary material for: RITA (Reactivating p53 and Inducing Tumor Apoptosis) is efficient against TP53abnormal myeloma cells independently of the p53 pathway
Source: BMC Cancer. 2014 Jun 14;14:437. doi: 10.1186/1471-2407-14-437 (PMC4094448; doi:10.1186/1471-2407-14-437)
Supplement: Additional file 2: Figure S2 — The true positive rates (sensitivity, Se) of the DR5 modulation (left) and the percentage of 17p deletion (right) were plotted in function of the false positive rate (1-specificity, 1-Sp). As indicated by the arrows, the respective 1.2 and 19% thresholds for the DR5 increase and 17p deletion provided 100% sensitivity and specificity. [file 1471-2407-14-437-S2.pdf]

Figure S2

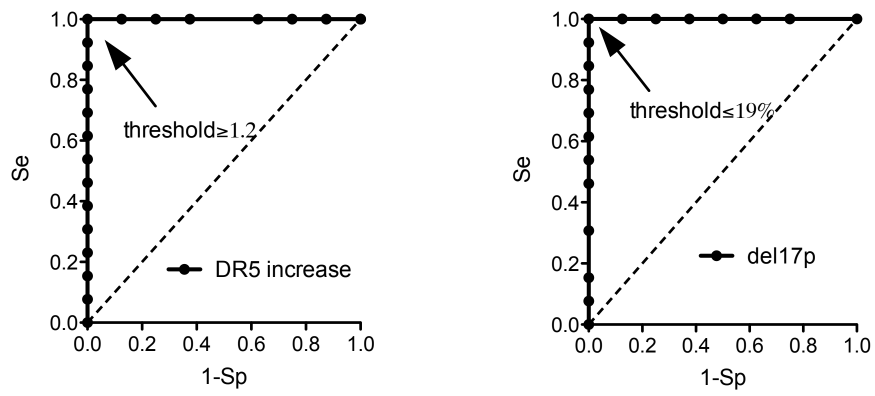

Figure S2. The true positive rates (sensitivity, Se) of the DR5 modulation (left) and the percentage of 17p deletion (right) were plotted in function of the false positive rate (1-specificity, 1-Sp). As indicated by the arrows, the respective 1.2 and 19% thresholds for the DR5 increase and 17p deletion provided 100% sensitivity and specificity.
